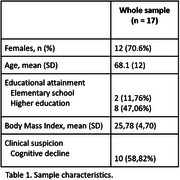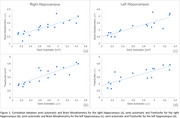# Automated and semi‐automated methods in quantitative and qualitative evaluation of the hippocampus

**DOI:** 10.1002/alz70856_106708

**Published:** 2026-01-08

**Authors:** Bruna Bressan Valentini, Andrei Bieger, Marco Antônio De Bastiani, Guilherme G. Schu Peixoto, Guilherme Povala, Diego Moraes Alves, Fernando Rigon, Artur Martins Coutinho, Laura Willers Souza, Mateus Rozalem Aranha, Marjana Reis Lima Rizzo, Eduardo R. Zimmer, Wyllians Vendramini Borelli

**Affiliations:** ^1^ Universidade Federal do Rio Grande do Sul, Porto Alegre, Rio Grande do Sul, Brazil; ^2^ Hospital Moinhos de Vento, Porto Alegre, Rio Grande do Sul, Brazil; ^3^ Masima: Macunaíma Soluções em Imagens Médicas, Porto Alegre, Rio Grande do Sul, Brazil; ^4^ Universidade Federal do Rio Grande do Sul, Porto Alegre, RS, Brazil; ^5^ masima: Macunaíma Soluções em Imagens Médicas, Porto Alegre, Rio Grande do Sul, Brazil; ^6^ University of Pittsburgh, Pittsburgh, PA, USA; ^7^ Center of Nuclear Medicine, Institute of Radiology, Hospital das Clínicas, Faculdade de Medicina da Universidade de São Paulo, São Paulo, São Paulo, Brazil; ^8^ Nuclear Medicine Section, Hospital Sírio‐Libanês, São Paulo, São Paulo, Brazil; ^9^ Sant Pau Memory Unit, Hospital de la Santa Creu i Sant Pau, Biomedical Research Institute Sant Pau, Universitat Autònoma de Barcelona, Barcelona, Spain; ^10^ Neuroradiology Section, Department of Radiology, Hospital de la Santa Creu i Sant Pau, Biomedical Research Institute Sant Pau, Universitat Autònoma de Barcelona, Spain, Barcelona, Spain; ^11^ Hospital Nossa Senhora da Conceição, Porto Alegre, Rio Grande do Sul, Brazil; ^12^ Federal University of Rio Grande do Sul (UFRGS), Porto Alegre, RS, Brazil; ^13^ Brain Institute of Rio Grande Do Sul, PUCRS, Porto Alegre, RS, Brazil; ^14^ McGill Centre for Studies in Aging, Montreal, QC, Canada; ^15^ Centro de Memória, Hospital Moinhos de Vento, Porto Alegre, RS, Brazil

## Abstract

**Background:**

The hippocampus is involved in the pathogenesis of several neurological diseases, including Alzheimer's disease (AD). AD‐related neurodegeneration commonly leads to a reduction in hippocampal volume, which can be assessed with Magnetic Resonance Imaging (MRI). This assessment can be performed using the medial temporal atrophy (MTA) scale and quantitatively through semi‐automatic and automated methods. The latter has been introduced recently but still not widely accessible in Brazil. Reproducibility among these methods is essential for clinical practice and monitoring of patients affected by the disease. In this study, we aimed to evaluate one semi‐automatic and two automated methods.

**Method:**

Hippocampal volume was evaluated using one semi‐automatic (SA) and two automated methods. MTA was evaluated visually by a radiologist and with automatic visual ratings of atrophy (AVRA). All evaluations were based on T1‐weighted volumetric MRI images, acquired using a 3T Spectra scanner (Siemens). The SA assessment involved hippocampal manual segmentation using a region of interest (ROI) approach and volume calculation within the manufacturer's software. Automatic methods included the manufacturer's Brain Morphometry (BM) software and FreeSurfer v7.4 (FS). Results were analyzed using Pearson's correlation coefficient and intraclass correlation coefficient (ICC), with the semi‐automatic method as the reference. MTA was assessed using z‐test with Bonferroni correction.

**Result:**

Seventeen participants were evaluated (Table 1). The findings showed a strong, positive, and significant correlation between SA and BM for the right hippocampus (r=0.84, *p* <0.001) and left hippocampus (r=0.86, *p* <0.001). Similarly, a strong, positive, and significant correlation was observed between SA and FS for the right hippocampus (r=0.85, *p* <0.001) and left hippocampus (r=0.86, *p* <0.001) (Figure 1). Data reliability tested with ICC showed excellent internal consistency between SA and BM for the right (0.90, *p* <0.001) and left (0.91, *p* <0.001) hippocampus, as well as between SA and FS for the right (0.90, *p* <0.001) and left (0.90, *p* <0.001) hippocampus. z‐test with Bonferroni correction showed no significant difference in MTA classification between methods (*p* <0.05).

**Conclusion:**

This preliminary study demonstrates a strong correlation between the methods. Fully automated analysis may enhance efficiency and help democratize the use of volumetric biomarkers in clinical practice, thereby better supporting the identification of AD‐related hippocampal atrophy.